# Impact of a Universal Nirsevimab Prevention Program Against Respiratory Syncytial Virus Bronchiolitis in Infants in Sicily (Italy) During the 2024–2025 Epidemic Season: A Retrospective Cohort Study

**DOI:** 10.3390/vaccines13121219

**Published:** 2025-12-02

**Authors:** Claudio Costantino, Emanuele Amodio, Rosario Asciutto, Costanza Affranchi, Franco Belbruno, Nicole Bonaccorso, Sonia Cilia, Fabio Massimo Contarino, Vincenzo Di Gaetano, Francesco Di Gregorio, Angelo Foresta, Roberto Furnari, Flavio Genna, Dario Genovese, Gabriele Giorgianni, Carmelo Massimo Maida, Sara Palmeri, Giovanna Parrino, Alessandra Piazza, Sebastiano Pollina Addario, Giovanni Tinervia, Fabio Tramuto, Francesco Vitale

**Affiliations:** 1 Department of Health Promotion, Mother and Child Care, Internal Medicine and Medical Specialties, University of Palermo, 90127 Palermo, Italy; claudio.costantino01@unipa.it (C.C.); flavio.genna@community.unipa.it (F.G.); dario.genovese@unipa.it (D.G.); carmelo.maida@unipa.it (C.M.M.); giovanni.tinervia01@unipa.it (G.T.);; 2 Sicilian Section of the Italian Society of Hygiene, Preventive Medicine and Public Health, 90139 Palermo, Italy; 3 PhD National Programme in One Health Approaches to Infectious Diseases and Life Science Research, Department of Public Health, Experimental and Forensic Medicine, University of Pavia, 27100 Pavia, Italy; 4 Department of Healthcare Activities and Epidemiological Observatory of Sicilian Region, Sicilian Health Department, 90100 Palermo, Italy; 5 Regional Reference Laboratory for Molecular Surveillance of Influenza, Clinical Epidemiology Unit, University Hospital “Paolo Giaccone”, 90127 Palermo, Italy

**Keywords:** respiratory syncytial virus, Nirsevimab, bronchiolitis, program impact, infant hospitalization, universal prophylaxis

## Abstract

**Objectives**: The introduction of universal prophylaxis with Nirsevimab represents a major innovation in preventing respiratory syncytial virus (RSV) infections in newborns. In Sicily, Nirsevimab administration began on 1 November 2024, for all newborns under one year and at-risk infants during the 2024–2025 season. This study assessed the real-world impact of this strategy in reducing RSV-related hospitalizations. **Methods**: This retrospective cohort study examined newborns residing in Sicily from 2015 to May 2025, evaluating hospitalization incidence rates attributable to RSV during the first year of life. RSV hospitalizations were identified using ICD-9 codes (079.6, 466.11, 480.1) in any diagnostic position. Incidence rates in the 2024–2025 season (intervention period) were compared with preceding seasons. **Results**: During the study period, 4431 RSV hospitalization cases occurred (19.84 cases per 1000 person-years), peaking in 2023–2024 (53.47 cases per 1000 person-years). A statistically significant 40% reduction in RSV hospitalizations was observed during the 2024–2025 season (October–April) compared with the preceding season, with a relative reduction ranging between 33.4% and 54.8% across sensitivity models. **Conclusions**: These results confirm the significant impact of the universal prophylaxis program in real-world practice, consistent with other European programs. These findings support universal RSV prevention strategies and provide insights for optimizing regional and national health policies.

## 1. Introduction

The respiratory syncytial virus (RSV) is an RNA pathogen belonging to the *Pneumoviridae* family and is recognized as the leading cause of lower respiratory tract infections in neonates and infants within the first year of life [1]. In the youngest patients, RSV infection can present with clinical manifestations of varying severity, ranging from mild, cold-like symptoms to severe complications such as bronchiolitis and pneumonia [2,3], with incidence peaking during the winter months. RSV is also one of the main causes of hospitalization in early infancy due to respiratory distress [4].

Beyond the acute phase, RSV infection is associated with long-term outcomes, particularly an increased risk of recurrent wheezing and asthma exacerbations later in childhood [5,6]. This emphasizes the urgent need for effective prophylactic strategies aimed at reducing the RSV-related disease burden during early infant life, which also have the potential to improve long-term pediatric respiratory health.

Globally, RSV is estimated to cause approximately 33 million episodes of lower respiratory tract infections in children under 5 years of age each year, leading to more than 3 million hospitalizations and about 120,000 deaths, primarily concentrated in low-income countries [7]. In high-income countries, RSV still imposes a substantial healthcare burden: the estimated incidence is 13.4 hospitalizations per 1000 children under 2 years of age, and 3–4 hospitalizations per 1000 in those under 5 years [8,9]. The incidence is particularly high among young infants (<6 months), ranging from 12 to 57 per 1000 [10]. Moreover, more than 60% of children contract at least one RSV infection within the first year of life, and almost all are infected by the age of two [11].

Until recently, the only available preventive option was Palivizumab, a short half-life monoclonal antibody indicated exclusively for high-risk pediatric subgroups, such as preterm infants < 29 weeks’ gestational age, children with hemodynamically significant congenital heart disease, or severe chronic lung disease [12,13]. However, the use of Palivizumab has been limited by several factors: high cost, the requirement for monthly administrations throughout the epidemic season, and restricted eligibility to a very small proportion of the pediatric population (approximately 4–6%) [14]. Given that prophylaxis with Palivizumab is limited to high-risk groups, the majority of RSV-related hospitalizations (70–90%) occur in otherwise healthy full-term infants who do not qualify for Palivizumab [15,16].

Since 2022, Nirsevimab has become available as a humanized long-acting monoclonal antibody targeting the RSV fusion (F) protein, an essential component for viral infectivity. Nirsevimab was approved in the European Union (31 October 2022) and in the United States (17 July 2023) [17,18]. Compared with Palivizumab, Nirsevimab is distinguished by its extended half-life, providing protection for at least 5 months with a single administration during the epidemic season, as well as by its potent neutralizing activity against a broad range of viral strains, including those resistant to other monoclonal antibodies [19].

Clinical trial data have demonstrated high efficacy: the phase 3 MELODY trial showed a 74.5% reduction in medically attended RSV-associated lower respiratory tract infections, with a ~77% reduction in hospitalizations [19]. The pragmatic HARMONIE trial further documented an 83.2% reduction in RSV hospitalizations and a 75.7% reduction in severe cases, with protection sustained for up to 180 days [20,21]. The safety profile of Nirsevimab has been consistently confirmed, with clinical studies reporting a low incidence of adverse events and no evidence of increased risk of serious events compared with placebo [22,23].

In light of these findings, several countries have launched universal immunization programs. Notably, the observational NIRSE-GAL study conducted in Spain during the 2023–2024 season showed that universal administration of Nirsevimab in infants significantly reduced RSV hospitalizations, with an estimated effectiveness of 84% against hospitalization and 90% against severe disease [24].

In Italy, the Nirsevimab immunization campaign began in 2024 following recommendations from the Ministry of Health, with priority given to newborns and infants entering their first epidemic season [25]. In Sicily, the administration of Nirsevimab started on 1 November 2024, and included all newborns under one year of age as well as those at risk during the 2024–2025 epidemic season.

Considering this innovation, the present study seeks to evaluate the real-world impact of this strategy during the 2024–2025 epidemic season, focusing on both hospitalization burden and disease severity.

Specifically, we hypothesized that the introduction of universal prophylaxis would lead to a measurable reduction in hospitalization rates, even in a transitional season characterized by incomplete and late-season coverage. Therefore, the main study objective was to assess the population-level impact of the universal prophylaxis program during the 2024–2025 season by analyzing the incidence of RSV-related bronchiolitis hospitalizations among infants born and residing in Sicily, and by comparing these data with those from previous epidemic seasons (2015–2024).

## 2. Materials and Methods

This is a retrospective cohort study conducted on children aged 0–59 months who were residing in Sicily from 2015 to May 2025, assessing the proportion of infants who experienced hospitalisations attributable to RSV with particular attention to newborns in their first year of life. The study was approved by the Ethics Committee of the “P. Giaccone” University Hospital in Palermo (Meeting Report No. 10/2022, dated 16 November 2022). Statistical analyses were performed using R (version 4.5.1).

**Figure 1:**
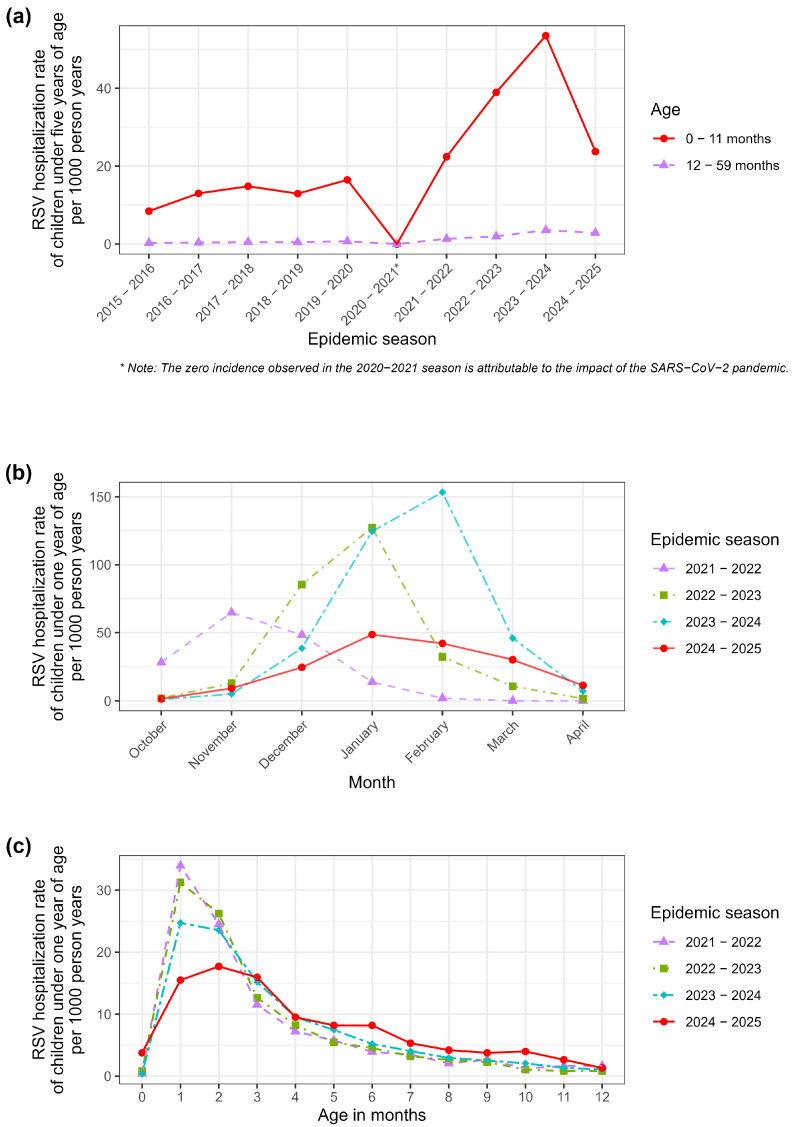
RSV hospitalization rates per 1000 person-years among children under one year of age. (**a**) Long-term trends (2015–2025) stratified by age group (0–11 months vs. 12–59 months). Note: The lower incidence prior to 2020 may reflect lower diagnostic sensitivity before the implementation of the RespiVirNet surveillance system. The zero incidence in 2020–2021 is attributable to SARS-CoV-2 pandemic restrictions. (**b**) Monthly distribution of hospitalization rates for the seasons 2021–2022 to 2024–2025, showing the shift in peak timing observed in the 2021–2022 season. (**c**) Age-stratified hospitalization rates (0–12 months) comparing the intervention season (2024–2025) with previous epidemic seasons.

Data were retrospectively retrieved from the Hospital Discharge Records (HDR) database of the Sicilian Region. This administrative database covers all public and private hospitals within the region and collects individual information on every hospital admission. The specific variables extracted for the analysis included demographic data (sex, month and year of birth, and province of residence), hospitalization details (date of admission, date of discharge), and clinical data including primary and secondary discharge diagnoses encoded using the International Classification of Diseases, 9th Revision, Clinical Modification (ICD-9-CM). Additionally, the Diagnosis-Related Group (DRG) weight was extracted and used as a proxy for clinical severity. The DRG relative weight represents a numeric value reflecting the average resources required to treat a patient in a specific DRG compared to the average hospital patient. Since higher resource consumption is associated with more intensive care and complex clinical management, the DRG weight is widely established as a valid proxy for disease severity [26]. It is important to state that the available dataset was aggregated and anonymized; therefore, it was not possible to link individual hospitalization events to the specific immunization status (Nirsevimab administration) of each infant. Consequently, the study was designed to evaluate the population-level impact of the universal prophylaxis program by comparing incidence rates across seasons, rather than measuring efficacy at the individual level. RSV hospitalizations were identified using specific ICD-9-CM codes in any diagnostic position: 079.6 (Respiratory syncytial virus), 466.11 (Acute bronchiolitis due to respiratory syncytial virus), and 480.1 (Pneumonia due to respiratory syncytial virus). The analysis included hospitalizations that occurred between October and April of each year, corresponding to the RSV epidemic season. The number of children born in each month and province, stratified by sex and epidemic season, was used as an offset variable. Specifically, incidence rates were calculated using “person-years” as the denominator. This metric represents the cumulative observation time contributed by the study population, calculated by summing the duration (in years) that each infant was at risk of hospitalization within the specific strata. The seasons prior to 2020–2021 (Figure 1a) were characterized by particularly low incidence rates, most likely due to limited diagnostic attention to the virus and the absence of a dedicated surveillance system prior to the full implementation of RespiVirNet, whose introduction significantly enhanced diagnostic sensitivity and improved the identification and reporting of RSV cases within the HDR database. It is also important to note that, prior to the standardization of surveillance systems (RespiVirNet), RSV testing practices in Italy were highly heterogeneous and often limited to severe clinical presentations, leading to a likely underestimation of the historical disease burden [27]. The 2021–2022 season (Figure 1b) exhibited an atypical epidemiological trend, characterized by an unusually early onset and a peak between October and November. This anomalous seasonality differed significantly from the traditional winter pattern. Since the 2024–2025 season returned to a standard seasonality (peaking in January–February), including the 2021–2022 season in the comparative analysis would have introduced significant temporal bias due to the mismatch in viral circulation dynamics. According to the previous considerations, inferential analyses were focused on the 2022–2023 and 2023–2024 seasons, which displayed epidemiological characteristics more representative of and comparable with those observed in the 2024–2025 season.

Four statistical models were fitted, assuming a Poisson Inverse-Gaussian distribution to account for overdispersion in the data, to evaluate the impact of the prophylaxis program introduced in the 2024–2025 season. The relative reduction in hospitalization rates was computed as (1 − IRR) × 100, where IRR represents the Incidence Rate Ratio. The first model compared the epidemic trend of the entire 2024–2025 season against the 2023–2024 season alone. The second model compared the epidemic trend of the entire 2024–2025 season against the aggregate of the 2022–2023 and 2023–2024 seasons. The third model compared the epidemic trend of the 2024–2025 season from January to April against the same months of the 2023–2024 season alone. The fourth model compared the epidemic trend of the 2024–2025 season from January to April against the same months of the aggregate of the 2022–2023 and 2023–2024 seasons. All models were adjusted for sex, province of residence, age in months and month of hospitalization.

The primary outcome of the study was the relative reduction in RSV-related hospitalizations observed during the 2024–2025 season compared with the 2023–2024 season. The apparent impact of the intervention was estimated as a proxy measure by calculating the percentage change in hospitalizations between the different seasons.

An additional question explored in this study concerned whether, beyond reducing the overall number of hospitalizations, immunization with Nirsevimab might also influence the clinical severity of disease among neonates hospitalized with RSV infection. To address this, the US weight of the Diagnosis-Related Groups (DRGs) was used as an indicator, as this parameter increases proportionally with clinical severity.

The universal immunization offer against RSV in the Sicilian Region with Nirsevimab followed a hospital-based delivery model similar to other European countries and Italian regions. Specifically, within 24–48 h of birth, in all Public and Private Sicilian Neonatology Units, the 50 mg Nirsevimab immunization was administered to “in RSV season” newborns (births included from 1 November 2024 to 31 March 2025). Moreover, the 100 mg Nirsevimab formulation was offered to all newborns born “out of season” from the 1 April 2024 to the 31 October 2024, starting from November 2024 at the Vaccination Centers of the nine Sicilian Local Health Authorities (Agrigento, Caltanissetta, Catania, Enna, Messina, Palermo, Ragusa, Siracusa, Trapani) or at the family pediatrician medical office. It should be noted that this universal prophylaxis program was implemented concurrently with the availability of the RSV maternal vaccine, which was offered to pregnant women at territorial vaccination centers during the same epidemic season.

At the end of the first immunization season against RSV the immunization coverage reached at the Regional Level among “in and out of season” newborns was between 65% and 70% (68.5%), below the immunization coverage rates reached in Galizia during the first immunization season [24].

## 3. Results

Between October 2015 and April 2025, a total of 4431 cases of RSV hospitalizations were documented in Sicily among children under one year of age (Table 1). Analysis of the seasonal distribution revealed variable trends. During the earlier seasons (2015–2019), the number of hospitalizations ranged on average between 200 and 300 cases per year, corresponding to hospitalization rates of about 10 cases per 1000 person years. The 2020–2021 season, coinciding with the SARS-CoV-2 pandemic, represented an epidemiological anomaly, characterized by a marked reduction in cases, primarily attributable to containment measures that limited the circulation of RSV and other respiratory viruses.

The peak incidence was observed in the 2023–2024 season, with 1106 bronchiolitis hospitalizations, corresponding to 53.47 cases per 1000 person years—the highest value across the entire observation period. In the following season (2024–2025), the first in which universal immunization with Nirsevimab was implemented, the number of hospitalizations decreased to 450 cases (23.73 cases per 1000 person years), with a markedly lower incidence compared with the peak of the previous year.

With respect to age distribution, most hospitalizations occurred in infants within the first three months of life (63.6 hospitalization cases per 1000 person years in the second month of life), the period known to carry the highest risk for RSV-related bronchiolitis. Hospitalization frequency progressively declined with increasing age, up to 11 months.

As shown in Figure 1a, in 2024–2025, there was a significant reduction in hospitalization incidence among children aged 0–11 months, but not among those aged 12–59 months.

In Figure 1b, the 2022–2023 and 2023–2024 seasons showed epidemiological characteristics more representative of and comparable with those observed in the 2024–2025 season and, thus, only these seasons were used for inferential analyses. This approach allowed for a more accurate comparison aimed at assessing the impact of the introduction of universal immunization with Nirsevimab in the neonatal population of the Sicilian Region. Comparing the current season with historical data requires careful consideration of the chosen baseline. As highlighted in Table 1, the overall hospitalization rate in the 2024–2025 season (23.73 per 1000) was significantly lower than the peak observed in 2023–2024, but remarkably similar to the rate recorded in the 2021–2022 season (22.41 per 1000). However, a closer inspection of age-specific incidence rates reveals a distinct epidemiological pattern (Figure 1c). In the 2021–2022 season, hospitalization rates were distributed relatively evenly across the first months of life. Conversely, in the 2024–2025 season—following the introduction of the universal prophylaxis program targeting newborns—a sharp divergence was observed. The incidence rate among infants aged 0–2 months (the primary target group for hospital-based immunization at birth) showed a marked reduction compared to both the 2023–2024 peak and the 2021–2022 baseline. In contrast, hospitalization rates among older infants (3–11 months), who were less likely to be intercepted by the catch-up campaign or were born outside the optimal window, remained elevated and comparable to previous high-circulation seasons.

**Figure 2:**
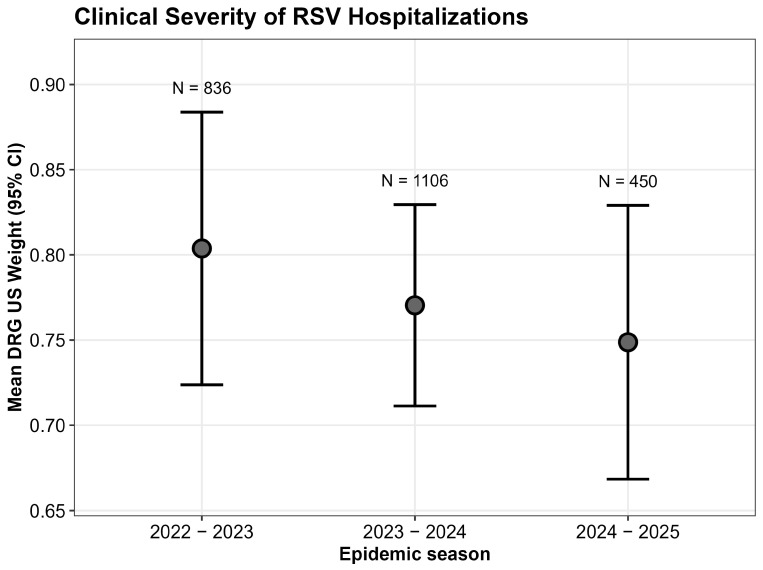
Clinical severity of RSV hospitalizations assessed by DRG (Diagnosis-Related Group) Weight. The plot displays the Mean DRG Weight (points) and 95% Confidence Intervals (error bars) for the reference seasons (2022–2023, N = 836; 2023–2024, N = 1106) and the intervention season (2024–2025, N = 450). No statistically significant difference in severity was observed across seasons (Kruskal–Wallis test, *p* = 0.9). DRG weights serve as a proxy for resource consumption and clinical complexity.

The analysis in Figure 2 shows that no statistically significant differences in terms of US DRG weight were observed across the 2022–2023, 2023–2024, and 2024–2025 seasons (Kruskal–Wallis test, *p* = 0.9) suggesting that infants who were hospitalized for RSV had similar severity of disease, regardless of the introduction of immunization.

**Figure 3:**
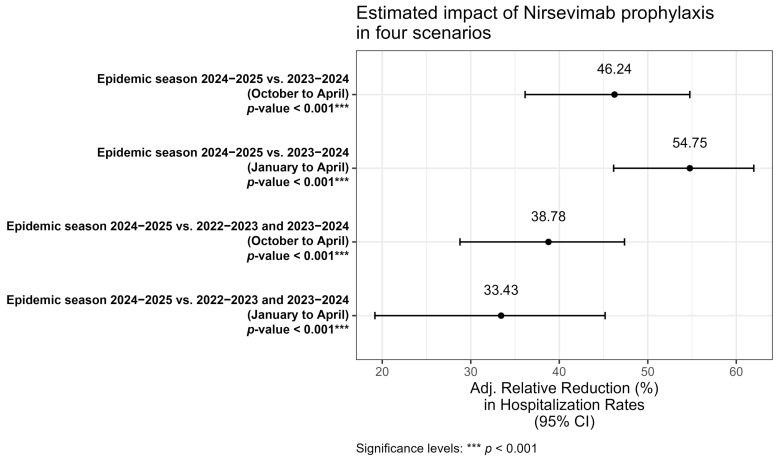
Adjusted Relative Reduction in RSV Hospitalization Rates. Forest plot showing the estimated impact of the universal prophylaxis program (1—Incidence Rate Ratio) expressed as a percentage reduction. The models compare the 2024–2025 season against different historical baselines (2023–2024 alone or the aggregate of 2022–2024) and time windows (October–April vs. January–April). Error bars represent 95% Confidence Intervals. All models are adjusted for sex, age in months, month of hospitalization, and province of residence.

To estimate the impact of the introduction of immunization with Nirsevimab (Figure 3), four multivariable models were developed to evaluate the relative reduction in hospitalization rates, adjusted for the same covariates (sex, age in months, and month of hospitalization).

In the first model (October–April 2024/2025 vs. 2023/2024) comparison between the entire 2024/2025 and 2023/2024 epidemic seasons showed a statistically significant relative reduction of 46.2%. In the second model (January–April 2024/2025 vs. 2023/2024), when restricting the analysis to the January–April period, the reduction increased to 54.7% while in the third model (October–April 2024/2025 vs. 2022/2023 and 2023/2024), when including two consecutive epidemic seasons as comparators, the reduction was 38.8%. Finally, a fourth model (January–April 2024/2025 vs. 2022/2023 and 2023/2024), restricting the analysis to January–April and comparing with the aggregate of the two preceding seasons, observed a reduction of 33.4%. Across all models, a statistically significant reduction in RSV hospitalizations was observed (*p* < 0.001), confirming the substantial impact of Nirsevimab introduction in the neonatal population. However, the magnitude of the reduction varied depending on the period considered and the comparator seasons.

## 4. Discussion

This study represents an important contribution to the evaluation of real-world evidence on the impact of universal prophylaxis with Nirsevimab in neonates. Our findings clearly indicate that the introduction of Nirsevimab resulted in an overall ~40% reduction in RSV hospitalizations during the 2024–2025 season (October–April) compared with the 2023–2024 season. While this confirms the positive program impact, the observed reduction may appear lower than that reported in other international contexts, as well as in the studies conducted in Spain documenting reductions exceeding 80% [24,28]. However, this discrepancy should be considered predictable, as organizational and temporal factors associated with the launch phase of the program in Sicily meant that only 50% of the target population was reached near the end of the epidemic season, and overall coverage of the target population did not exceed 70%. Consequently, a substantial proportion of the target population entered the beginning of the epidemic season without optimal coverage, thereby limiting the overall impact of the intervention. Of course, the estimated relative reduction represents an ecological proxy rather than a direct measure, as HDR data did not allow for identification of the immunization status of hospitalized infants and, thus, estimates could change according to the season considered as referent. Therefore, residual confounding with an impact on estimated rates cannot be excluded, such as differences in viral circulation intensity between the 2024–2025 and 2023–2024 seasons.

A critical aspect of interpreting these findings lies in the selection of the baseline season. We acknowledge that the estimated impact of the program is highly sensitive to the comparator year. The 2023–2024 season, used as the primary reference in our models, represented an exceptional epidemiological event characterized by the highest recorded incidence of RSV hospitalizations (53.47 per 1000). This surge was likely driven by the “immunity debt” phenomenon resulting from pandemic-related non-pharmaceutical interventions, which led to an expanded pool of susceptible infants and off-season outbreaks [29]. Comparing the 2024–2025 season against this peak inevitably yields a high efficacy estimate. Conversely, if the 2021–2022 season (22.41 per 1000) were selected as the baseline, the overall reduction would appear negligible. However, assuming that the 2024–2025 season merely reflects a natural return to pre-peak levels would be an ecological fallacy. If the reduction were solely due to a natural decrease in viral circulation, a uniform decline across all age groups would be expected. Our data, however, demonstrate a strong age-dependent effect. The reduction in hospitalizations was disproportionately concentrated in neonates aged 0–2 months, who benefited from high immunization coverage at birth. Meanwhile, older infants, who represent the “unprotected” or “lower-coverage” fraction of the cohort, continued to experience hospitalization rates consistent with high-circulation years. This age-stratified divergence provides compelling evidence that the universal prophylaxis program exerted a protective effect specifically where it was applied, effectively mitigating what might have otherwise been another high-impact season for neonates.

Beyond impact estimates, our study provides further insights into both the general epidemiology of RSV and the characteristics of subjects who experience hospitalization. Regarding the former, it appears evident that before 2020, hospitalisations for RSV showed lower incidence rates compared with the subsequent period. It is likely, however, that this finding is largely attributable to substantial underreporting, due both to the fact that before 2020 the national surveillance system for respiratory syndromes (INFLUNET) did not include RSV as in the case of the new surveillance system (RESPIVIRNET), and to the progressive availability of new diagnostic technologies that have facilitated and expanded the etiological identification of microorganisms responsible for respiratory syndromes, such as RSV bronchiolitis. Furthermore, it cannot be excluded that the marketing authorization of both monoclonal antibodies against RSV and RSV vaccines has increased awareness among healthcare professionals, thereby improving performance in terms of ICD-9-CM coding.

It is also worth noting that the hospitalization rate observed in the 2024–2025 season (23.73 per 1000) returned to levels similar to those recorded in 2021–2022 (22.41 per 1000). This similarity raises the question of whether the observed reduction compared with the 2023–2024 peak might be partly attributable to a natural epidemiological “readjustment” following the post-pandemic “immunity debt” surge. However, unlike the 2021–2022 season, the 2024–2025 reduction was not uniform across all ages. As shown in our results, the decline was predominantly driven by infants aged 0–2 months (Figure 1c), whereas older infants did not show a proportional decrease. This age-specific pattern strongly suggests that the reduction was directly associated with the administration of Nirsevimab in the birth cohort rather than solely due to natural fluctuations in viral circulation.

An additional consideration concerns the severity of RSV-related illnesses in hospitalized children, which in our study does not appear to differ in 2024–2025 compared with previous seasons. In contrast, pivotal clinical trials suggested that immunization could also have an impact on disease severity. Nevertheless, our findings may not be inconsistent with trial results for two main reasons. First, DRG attribution is primarily tariff-based and is not designed to capture clinical severity with sufficient accuracy. Secondly, since immunization coverage reached approximately 70%, the infants hospitalized in 2024–2025 likely represent the non-immunized fraction of the population. Consequently, it is expected that their severity profile would mirror that of patients in seasons when monoclonal antibodies were not available. Therefore, their clinical presentation and disease severity remained consistent with historical patterns, confirming that in the absence of protection, the clinical burden on the individual patient is unchanged. Last but not least, recent observational studies have highlighted that reductions in hospitalization rates do not necessarily translate into decreases in the clinical severity of cases requiring admission [30].

Moreover, our study also highlights that infants in the first three months of life are at the highest risk of hospitalization, making this group the population in which immunization presents the most favorable cost-effectiveness profile and on which maximum effort should be focused to achieve the highest possible coverage. However, in 2024–2025, the main reduction in hospitalization incidence was attributable to the 1–2 month age group, whereas in other age groups the risk remained relatively stable compared with previous seasons. This suggests that RSV circulation was still substantial in 2024–2025, but in infants aged 1–2 months, it resulted in a significant reduction in hospitalizations.

Naturally, although our study has a cohort design, its assessment of impact is ecological, as we do not have information on how many of the hospitalized neonates were actually immunized. Unfortunately, this represents the main limitation of the study, a limitation shared by much of the available literature on the subject, particularly during these early years of evaluating program effectiveness. From a methodological perspective, several observational studies have emphasized the importance of integrated surveillance systems capable of distinguishing between immunized and non-immunized populations to improve the precision of effectiveness estimates [31]. The availability of individual-level data, including immunization status, would allow for more accurate evaluation of the impact of prophylaxis not only in terms of hospitalization reduction but also regarding long-term outcomes, such as the risk of recurrent wheezing and pediatric asthma [4,5,6]. It is therefore crucial to implement integrated surveillance systems capable of combining clinical and immunization data at the individual level, in order to measure more precisely the real-world effectiveness of RSV immunization and to provide stronger support for future prevention strategies [32].

Additionally, the study is limited by the instability of the historical baseline used for comparison. The RSV seasons preceding the program were heavily influenced by the SARS-CoV-2 pandemic, resulting in irregular seasonality and magnitude (e.g., the immunity debt peak of 2023–2024). This variability makes it challenging to establish a “standard” pre-intervention incidence rate. While we addressed this by analyzing age-specific trends to isolate the intervention’s effect, the lack of a stable, multi-year pre-pandemic baseline remains a constraint in quantifying the exact magnitude of the reduction.

Furthermore, the extracted dataset did not include specific clinical variables such as ICU admission rates or the use of mechanical ventilation, which would have provided a direct measure of clinical severity. However, since the DRG system assigns higher weights to cases requiring complex procedures (including respiratory support), the analysis of DRG weights serves as an aggregate, albeit indirect, proxy for overall disease severity.

Moreover, reliance on administrative ICD-9-CM codes without direct linkage to virological results poses a potential risk of misclassification bias. Some hospitalizations might have been attributed to RSV without confirmation, while others could have been missed. However, in the Sicilian Region, the diagnostic workflow for respiratory viruses is highly centralized at the Regional Reference Laboratory for Molecular Surveillance (University Hospital “Paolo Giaccone” of Palermo). This centralization ensures uniform high-quality testing standards and consistent diagnostic protocols across the territory. Therefore, we are confident that this organizational structure significantly minimizes the inaccuracy related to diagnostic variability, making the potential impact of misclassification bias on our study results likely negligible.

Finally, we performed a preliminary and exploratory economic estimation. Considering that in 2022–2023 and 2023–2024 RSV hospitalizations generated average costs of €2,905,673, applying a relative reduction of 54.75% (best-case scenario) would have resulted in potential savings of €1,590,856. Conversely, with a relative reduction of 33.43%, the reduction in costs would have amounted to €971,366. However, these figures must be interpreted with caution, as they represent broad projections based on average tariffs rather than a precise cost–benefit analysis. Future studies with granular economic data are required to verify and refine these potential savings.

Overall, the previously reported limitations could clearly reduce the accuracy of the estimates; nevertheless, we believe that our study still plays a role in confirming that the introduction of Nirsevimab in Sicily has produced a significant and consistent impact in reducing RSV-related hospitalisations, with an immunization coverage of 65–70% in the cohort targeted during the first epidemic season. Our results confirm that, despite epidemiological differences between reference seasons and delays in campaign initiation, the intervention exerted a relevant public health impact, consistent with evidence from other universal immunization programs implemented in Europe [24,28].

## 5. Conclusions

Looking forward, the Sicilian experience is part of a broader transition toward universal RSV preventive strategies already underway in several European countries [24,28]. Specifically, our results suggest that the hospital-based delivery immunization model, administered at birth centers before discharge, is highly effective in ensuring high adherence and immediate protection for the most vulnerable age group (0–2 months). Therefore, regional and national health policies should prioritize integrating Nirsevimab administration into routine neonatal care pathways to maximize coverage and reduce the burden of early-onset disease. The consolidation of such programs may contribute not only to reducing the immediate burden of hospitalizations but also to improving long-term respiratory outcomes in children [5,6,33]. However, it is important to emphasize that Nirsevimab represents a passive immunization strategy with a likely limited duration over time; consequently, other active immunization strategies might be necessary to integrate with this strategy to ensure sustained protection. At the same time, the advent of vaccines specifically targeting pregnant women and older adults [4,34] opens the perspective of cross-protection for the most vulnerable groups. Nevertheless, the Sicilian experience suggests that integrating these strategies requires overcoming significant operational complexities. Specifically, we observed challenges in tracking maternal status to avoid redundant administration and noted lower adherence to vaccination during pregnancy due to hesitancy compared to the high acceptance of infant prophylaxis. Thus, while active vaccines are crucial for long-term sustainability [4,32], direct newborn immunization currently offers a more immediate and reliable coverage solution.

## Figures and Tables

**Table 1:** Hospitalization cases and rates per 1000 person-years by epidemic season (2015–2025), sex, and age in months. * Note: The zero incidence observed in the 2020–2021 season is attributable to the impact of the SARS-CoV-2 pandemic.

Variable		Hospitalization Cases	Person Years	Rates × 1000 Person Years
Overall		4431	223,345	19.84
Epidemic season	2015–2016	204	24,163	8.44
	2016–2017	316	24,300	13
	2017–2018	360	24,280	14.83
	2018–2019	304	23,494	12.94
	2019–2020	372	22,584	16.47
	2020–2021 *	0	21,843	0
	2021–2022	483	21,555	22.41
	2022–2023	836	21,480	38.92
	2023–2024	1106	20,683	53.47
	2024–2025	450	18,963	23.73
Sex	F	2105	108,282	19.44
	M	2326	115,063	20.22
Age in months	0	333	17,967	18.53
	1	1196	18,806	63.6
	2	908	19,379	46.86
	3	598	19,876	30.09
	4	385	19,760	19.48
	5	276	19,530	14.13
	6	213	19,033	11.19
	7	157	18,546	8.47
	8	127	17,884	7.1
	9	91	17,711	5.14
	10	75	17,399	4.31
	11	72	17,454	4.13

## Data Availability

The data that support the findings of this study are not publicly available as they were obtained from the Sicilian Regional Health Department. Data may be obtained upon reasonable request from the Sicilian Regional Health Department.
